# DC Power Line Communication (PLC) on 868 MHz and 2.4 GHz Wired RF Transceivers

**DOI:** 10.3390/s22052043

**Published:** 2022-03-05

**Authors:** Vlad Marsic, Tazdin Amietszajew, Petar Igic, Soroush Faramehr, Joe Fleming

**Affiliations:** Centre for Advanced Low-Carbon Propulsion Systems, Institute for Clean Growth and Future Mobility, Coventry University, Coventry CV1 5FB, UK; vlad.marsic@coventry.ac.uk (V.M.); taz.amietszajew@coventry.ac.uk (T.A.); petar.igic@coventry.ac.uk (P.I.); soroush.faramehr@coventry.ac.uk (S.F.)

**Keywords:** power line communication, 868 MHz, 2.4 GHz, Li-ion, battery, smart cell

## Abstract

Efficient management through monitoring of Li-ion batteries is critical to the progress of electro-mobility and energy storage globally, since the technology can be hazardous if pushed beyond its safety boundaries. Battery management systems (BMSs) are being actively improved to reduce size, weight, and cost while increasing their capabilities. Using power line communication, wireless monitoring, or hybrid data links are one of the most advanced research directions today. In this work, we propose the use of radio frequency (RF) transceivers as a communication unit that can deliver both wired and wireless services, through their superior analog and digital signal processing capability compared to PLC technology. To validate our approach computational simulation and empirical evaluation was conducted to examine the possibility of using RF transceivers on a direct current (DC) bus for wired BMS. A key advantage of this study is that it proposes a flexible and tested system for communication across a variety of network scenarios, where wireless data links over disrupted connections may be enabled by using this technology in short-range wired modes. This investigation demonstrates that the IEEE 802.15.4-compliant transceivers with operating frequencies of 868 MHz and 2.4 GHz can establish stable data links on a DC bus via capacitive coupling at high data rates.

## 1. Introduction

### 1.1. Health Monitoring Technology in Energy Storage

Li-ion rechargeable batteries are the technology behind current progress in electric mobility and the development of stationary energy storage systems. Delivering a superior performance when compared to different battery chemistry is a challenging task; stress factors, such as temperature, pressure, vibration and overcharging, may lead to hazardous runaway reactions inside the cells when they exceed the safe usage threshold. To ensure the safety of large-scale systems developed for power transportation platforms or store renewable energy, various battery management systems (BMSs) are being designed and implemented [1,2]. Since battery monitoring and control technology augments the power delivering system, reducing the weight, complexity, and expenses introduced by additional wiring is the current focus in BMS research and development. Furthermore, applying in situ monitoring through innovative sensor integration at cell level [3,4] for battery state-of-health (SOH) diagnosis [5,6,7] and safety, such as preventing thermal runaway [8,9], is the current state of action towards the smart cell [10,11].

**Figure 1:**
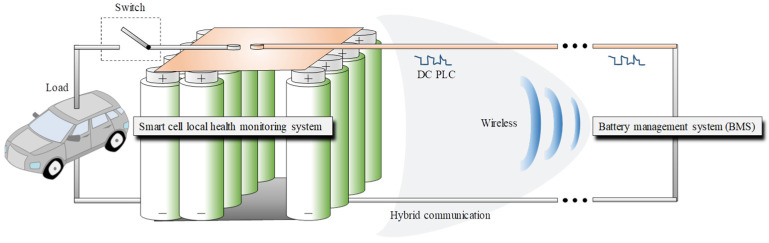
Battery monitoring system (BMS) research objectives.

The state-of-the-art BMS technology is concentrated on power line communication (PLC) [12,13,14], wireless monitoring [15,16], and hybrid techniques combining both methods [17]. Providing a stable communication link with a local management system located in close proximity to the cell may result in faster charging for current Li-ion technology [18,19] or, alternatively, a temperature-controlled environment is required by the new fast-charging cells [20]. Figure 1 illustrates the data service’s objective of the future BMS technology in anticipation of a revolutionary electric mobility that meets the highest level of security and potentially provides autonomy to transporting vehicles [21,22].

### 1.2. Proposed Solution

**Figure 2:**
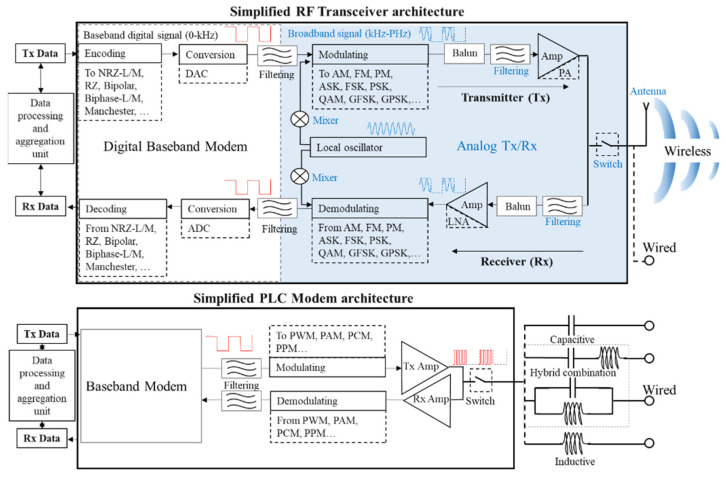
Internal architecture schematic representation for a generic RF transceiver (**top**) and a modem adapted for power-line communication (PLC) purposes (**bottom**). While the PLC modem can be connected to a network through inductive, capacitive, or hybrid wired coupling, the RF transceiver possesses an additional coupling via its antenna for wireless interconnections.

Since PLC technology is currently separate from wireless technology, which are both under development in order to meet the data exchange requirements of the power storage environment, in this work, we propose the use of RF transceivers as communication units that can deliver both wired and wireless services. The advantages over modem systems are various, and Figure 2 outlines the internal architecture similarities and differences between the two technologies. Contrary to the RF transceiver, which includes a baseband modem to interface with its high frequency radio module [23,24], the PLC makes use of previously existing technologies [25,26], adapts the current Ethernet, LIN, CAN controllers [27,28,29,30,31], or emulates software modems on microcontroller platforms [32,33,34]. It is important to note that RF transceivers are different from digital modems in that RF transceivers have to filter the digital signal by an analog output in order to comply with EMI regulations regarding spurious emissions generated during switching at high frequencies.

RF transceivers are also advantageous due to their high Rx sensitivity developed when low power functioning is a requirement. Additionally, since RF transceiver technology primarily operates on high frequency spectrum compared to modems, it is only necessary to add a capacitive coupling element, such as an antenna to a short physical wired network [35] or the antenna and a quarter wavelength coaxial link acting as an proximity impedance transformer [36,37].

**Figure 3:**
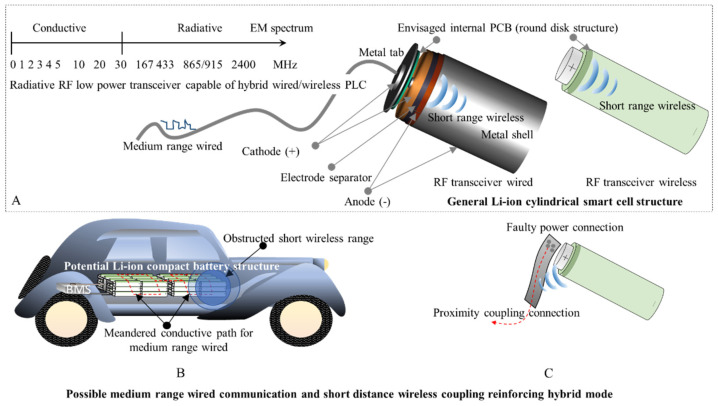
Illustration of the potential advantage of exploiting a short wireless range by inserting an RF transceiver inside a cylindrical metal shell with communication modules connected through physical wires instead of antennas. The general internal smart cell structure (**A**) is exemplified in a potential battery structure where the wireless obstructed short range is compensated by wired links (**B**), while a faulty power connection may still be bridged via RF coupling (**C**).

In comparison to the wired format proposed for the RF transceivers in this study, the wireless format may offer increased link budgets when nodes are appropriately spread. However, in high density scenarios inside a highly reflective environment, such as in a battery composed of thousands of cells, a wired conductive is more advantageous. The meandered battery’s metal maze may be untangled by using wires as leaky waveguides, similar to the trials for AC PLC that demonstrated a greater wireless range close to the power cables [35,36,37], when high frequency radio signals are transmitted using wires as leaky waveguides. In addition, if the radio transmitter unit must be embedded inside a metal case, as is the case with cylindrical lithium-ion batteries, then, again, the cabled option may have an advantage for short cable networks. Furthermore, the high frequencies, such as 868 MHz and 2.4 GHz, suffer from strong attenuation and losses from connection and insertions, so the potential radiation may reach sub-meter ranges, insufficient to solve the hidden node problem, but enough to overcome a faulty tab connection in a hybrid wired–wireless mode. Figure 3 illustrates the advantages of high frequency radio transceivers wired for PLC versus traditional wireless data links.

Although most of the developed PCL modems operate on carriers up to 30 MHz to match the impedance of long power cables [38] and avoid interference with local area networks (LANs) and smart metering [39,40], advantages of higher frequencies to increase data rate or signaling are also considered [41,42]. The segment of the electromagnetic spectrum (EM), from 1 up to 30 MHz, is classified as conductive from the EM compatibility (EMC) point of view according to the ETSI standard [43], the upper band being associated with the radiative EM. As the transceiver technology proposed for testing in this study uses the 868 MHz and 2.4 GHz, its signal carriers are in the radiative range, therefore short wires attached to the modules enable a wired–wireless hybrid operating mode.

The present study focuses on the use of well-established robust technologies operating at high frequencies (HFs) of 868 MHz and 2.4 GHz. As part of an analysis of whether these transceiver technologies can deliver a stable data link over a short wired PLC environment, such as the one encountered in battery packs, a simulation followed by empirical experiments is conducted. The investigation metric is the received signal strength (RSS) for the simulation or the received signal strength indicator (RSSI) for the empirical test, in combination with the potential bit error rate (BER) or packet error rate (PER) indicators. The communication system is capacitively connected to the DC bus composed of four 18650 Li-ion cells presenting a state of charge (SOC) of 100%. Experimentally, the pros and cons of the two proposed HF frequencies of 868 MHz and 2.4 GHz are determined by varying payload size and coupling capacitance. The receiver (Rx) sensitivity of approximatively −100 dBm, such as the ones delivered by the TI transceivers used in the experiment, −109 dBm for CC1200 [44], and 99 dBm for CC2520 [45], are used as a reference for the data link available budget, while the transmitter (Tx) power is set at the generic value of 0 dBm.

### 1.3. Contribution and Paper Organization

By proposing the use of RF transceivers as replacements for DC PLC modems in short wired networks, such as those in Li-ion battery mobile platforms, we can benefit from the wide availability of various signal modulations and data rates on robust high sensitivity systems, instead of dealing with the limitations of modem technology. Furthermore, since the wired network can function as an antenna-leaky cable, physical damage to connections may be overlooked as hybrid wired/wireless functionality, which could lead to greater opportunities for analysis and diagnostics rather than investigating the causes of the loss of connectivity. Since measurement redundancy ensures safety and prevents failure or drift over time, the research presented in this paper delivers an important contribution to BMS research by illustrating the possibility of using the RF transceivers for wired PLC, whereas they are typically associated with wireless communication. With this presented application, future smart cells equipped with RF transceivers may be able to provide hybrid BMS data services as well as diagnose fault connections within cell networks.

The work presented in this article is organized in four main sections. As the Introduction explains the background and motivation for the study, the second section covers the methodology employed in the investigation; the experimental testing and pre-sets are prefaced by a simulation, which delivers the complementary data regarding the network equivalent circuit as a system reacting to tailored input resulting from priory measurements. A discussion is provided about the potential implications of data variation in section three, based on simulations and empirical results. The final section summarizes the findings and highlights the potential benefits that may result from pursuing the avenues newly opened by this study.

## 2. Methodology and Experimental Setup

The methodology of testing wired communication via RF transceivers running at 868 MHz and 2.4 GHz for DC PLC involves two stages: preliminary circuit measurements and simulation, experimental testing and data recording for various payload sizes at maximum data rate allowed by the IEEE 802.15.4 standard for those frequencies. Since it is common knowledge that at low data rates the data signal is more noise resilient and therefore it offers a better communication stability, the DC PLC experiment is set to investigate the 150 kbps for 868 MHz and 250 kbps for 2.4 GHz to determine if these high data transfers may be exploited on a common power line connected to four 18650 Li-ion cells arranged in parallel and then in series, whereas at 100% SOC. The metric for a stable connection is provided by the RSSI and the transmission errors BER and PER. It is expected that for a stable connection, the RSSI is under −50 dBm, while BER and PER is 0 since variable loads or significant noises are not included in this proof-of-concept experimental stage.

### 2.1. MATLAB Simulation Setup

To proceed with the wired high frequency communication simulation scenario, two factors need to be accounted for: the minimum input parameters setting the simulation boundaries and initial conditions, and a dedicated electrical simulation environment. Since the experimental platform delivered by the TI evaluation RF kit SMARTRF TRXEBK [46], including the wireless transceivers on 868 MHz CC1200 [47] and on 2.4 GHz CC2520 [48], was available, some signal attributes, such as the amplitude at 0 dBm, transmitting power were straightforward to be determined. Moreover, as the common modulation scheme for both distinct TI transceivers supporting the standard data rates of 150 kbps and 250 kbps at 868 MHz and 2.4 GHz, respectively, is a Gaussian frequency shift keying modulation (GFSK), the amplitude measurement was performed for this modulation scheme. 

The acknowledged scheme for the GFSK modulation builds on a Gaussian filter applied to digital data, followed by an integrator to split the signal into its trigonometric quadrature I/Q components, which is mixed with the high frequency carriers and summed at the output to deliver the frequency shift keying (FSK) scheme [49,50,51,52]. Nevertheless, implementing the GSFK modulation from other schemes, such as continuous phase modulation (CPM) [53,54] or the Gaussian minimum shift keying (GMSK) [55], or applying smoothing after FSK [56] is becoming increasingly popular due to the advances in computer simulation platforms. In this study, for simplicity, a generic GFSK signal transmitter, using the amplitudes previously measured with the oscilloscope, is implemented accordingly to each of the two communication frequencies [55], sending a random binary message over a 20 MHz bandwidth (i.e., central carrier frequency ±10 MHz) continually. The wide bandwidth was adopted to exceed Nyquist and Shannon’s minimum necessary for a 1 Mbps bitrate, which is higher than the experimental envisaged 150 and 250 kbps and for a better visibility of the frequencies in the spectrum measurement.

For the signal amplitude measurement, the R&S RTM3004 oscilloscope [57] was used. The TI kit’s daughter boards equipped with CC1200 and CC2520 transceivers were subsequently connected to the measurement equipment thought a 50 Ω coaxial cable of 1 m length via sub miniature version A (SMA), whereas the motherboard interfaced trough a USB cable was linked to a control PC running TI’s SmartRF Studio 7 [58]. Each transceiver board was set individually to transmit a payload of 120 bytes indefinitely at 0 dBm using a GFSK modulation, at 150 kbps for 868 MHz and 250 kbps for 2.4 GHz. The signal’s peak-to-peak amplitude recorded a value of 383 mV for 868 MHz and 413 mV for 2.4 GHz. Since the measurement is useful in a further electrical simulation of a signal source or transformed in RMS to reference a power setting model, the focus was directed towards the selection of a simulation platform, capable of various calculations in the time and frequency domains.

**Figure 4:**
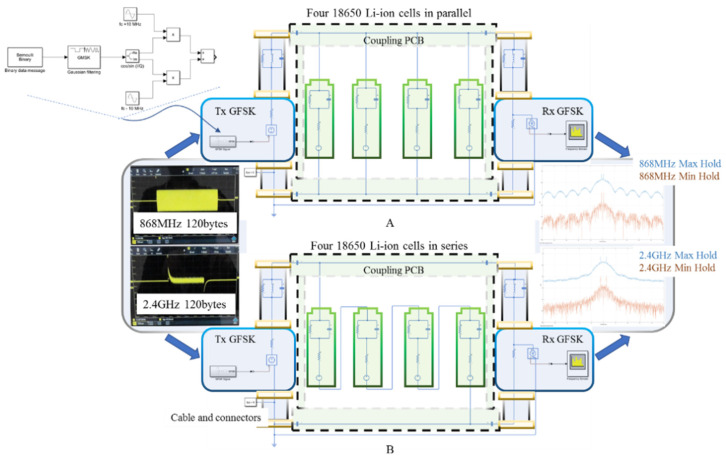
MATLAB Simscape test setup for 868 MHz and 2.4 GHz communication over the DC bus composed of four Li-ion cells organized in parallel (**A**) and series (**B**).

MATLAB was selected for the simulations for its multiple simulation options, from software coding to the usage of predefined integrated solvers in its visual programing environment Simulink. Since an integrated virtual electrical schematics simulation reduces the possible errors introduced through a potential challenging software coding, the Simulink’s Simscape toolbox was selected to deploy the testing of the wired high-frequency communication scenario. Figure 4 schematically illustrates the implemented Simscape schematics for four 18650 Li-ion cells connected in parallel A and in series B. The generic parasitic resistance, R, inductance, L, and capacitance, C, of the cables and connectors were accounted for through a symmetrical parallel RLC element at both schematic ends. While the MATLAB Simscape’s integrated battery models or circuit elements included in the Power Electronics toolbox present an attractive option of simplifying the schematics and reduced simulation parameters, in high frequency testing they display a less-transparent behavior. Moreover, since the toolbox’s basic elements are modeled as current and voltage sources, they also restrict the parallel and series component’s intuitive connections; therefore, the Simscape Foundation Library electrical component’s alternative is used in this model. Each cell is generically represented by a DC power source limited by a serial resistor in series with a parallel RC element, as described in the classical Thevenin battery model [59]. The GFSK receiver is represented by a virtual spectrum analyzer, reading the output signal feed to a 50 Ω resistor that mirrors the RF transceiver internal impedance. As the capacitive coupling is the straightforward interfacing method in PLC, the plus and minus lines were both connected to the Tx and Rx through capacitor sets of 1 µF, 1 nF and 1 pF, to determine the best high-frequency behavior for 868 MHz and 2.4 GHz.

### 2.2. Empirical Experimental Setup

The TI evaluation RF kit TRXEBK is the choice platform for the experimental wired DC PLC at high frequencies, since it provides a common software and hardware environment for both interest communication frequencies of 868 MHz and 2.4 GHz, ensuring consistency at the empirical investigation stage. Two PCs were used to control and manage the transmitter and receiver via a USB cable trough the TI’s SmartRF Studio 7 dedicated interface. The SMA output of each transceiver was connected through a 50 Ω coaxial cable, i.e., the same was previously used to connect the R&S oscilloscope to a custom prototyping PCB board designed for the DC power network coupling.

The coupling to a network for transmitting and receiving data through and from a physical signal is mainly based on impedance matching principles, such as for optimal power transfer to a load or for minimum noise [60]. Neither of the methods come without criticism [61,62]. Additionally, regardless of the impedance circuitry used for coupling and recently applied communication standards [63,64], the signal injected for PLC encounters multiple reflections, attenuations and additive noises, resulting from unplanned parameters, such as the cable branches’ unknown connection state, the random coupling of loads and various impulsive interferences [65,66]. In the case of the DC power bus bound by Lithium-ion energy storage, such as in this study, simplifications of coupling circuits can be achieved for complex network measurements [12,67] and communication signal injection [25,26,68,69] through capacitive connections.

**Figure 5:**
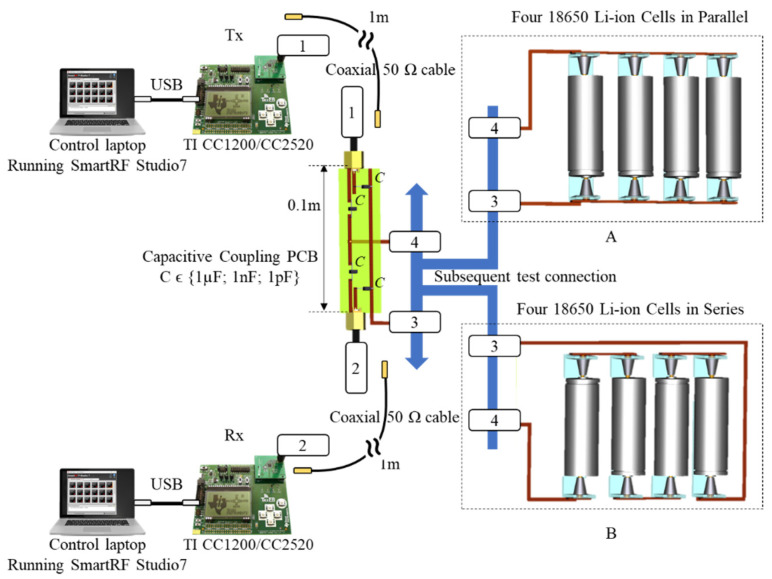
Experimental test setup for 868 MHz and 2.4 GHz communication over the DC bus composed of four Li-ion cells organized in parallel (**A**) and in series (**B**).

This simplification exploits the capacitive property of buffering the DC voltages, while presenting transparency to the AC signals; however, due to the various number of unknown factors that may play a role during communication, an empirical test for different capacitive values may provide a more realistic picture of possible communication measures and adjustments. Following the above reasoning, three identical boards were made, each presenting four different coupling capacitors, 1 µF, 1 nF and 1 pF, similar to those used for the MATLAB simulation. The capacitive coupling PCB is subsequently connected via banana-ended cables to the four 18650 Li-ion cells arranged in parallel and then in series. Each cell presents 100% SOC and it presents a nominal voltage of 4.2 V and 3500 mAh capacity. Figure 5 illustrates the setup and the general arrangement and device interconnections.

The empirical measurements include two more additional setups than in the MATLAB simulation, to complete the picture of using high-frequency RF transceivers with DC PLCs. Since both CC1200 and CC2520 transceivers are used in wireless applications, a setup testing the possible wireless connection between the Tx and Rx in a straight line separated by 2 m cables and 0.1 m distance (i.e., the PCB size) between the cables’ ends was investigated to, first, reference the worst communication case scenario, i.e., if the PCB and the batteries were removed while the cables and transceivers were still in place and running. A second setup, connecting the end cables to the interfacing PCBs without adding the Li-ion cells and holders to the scenario was designed to test the best communication case scenario, when the voltages, currents and additional circuit parasitic elements are missing. The two additional setup roles present potential reference frames for the tested DC PLC, the disconnection, and only the PCB connection, providing the overall operating margins that, in theory, may be described by RSSI, BER and PER.

Across all four empirical setups, the Tx–Rx data link was tested with three different payload sizes of 10, 60, and 120 bytes. It is designed to investigate whether major differences can be detected at 150 kbps for 868 MHz and 250 kbps for 2.4 GHz, when the transmission power is 0 dBm, while a variety of size data is exchanged.

## 3. Results and Discussion

For each 10 µs simulation time delivered through a local Backward Euler solver with a sampling period of 10 ps, the MATLAB simulation runs for approximately 1 min. The results are recorded with the virtual spectrum analyzer on the communication central frequency peaks using the maximum and minimum hold trace option. This method delivers the maximum and minimum received signal strength (RSS) for the simulated GFSK signal. On the experimental side, 100 consecutive frames transmitted at 0 dBm are measured through their RSSI, BER and PER. It needs to be noted that for the four designed experimental setups, the BER and PER were zero, therefore the only transmission metrics were provided by the RSSI.

To illustrate the RSS and RSSI for different coupling capacitances alongside the graph’s markers showing the average value, the error bars are used to illustrate the RSS or RSSI highest recorded value (the bar’s upper end) and lowest peak (the bar’s lower end). 

Consequently, the error bars show experimental data intervals’ minimum and maximum ends [70], although referring to them as the min–max range bars [71,72] would be more clear and therefore it will be used in describing this study’s results. The dashed line shows a possible linear relationship between the individual measurements and it was plotted to outline the value’s association with a communication frequency or the other.

### 3.1. MATLAB Simulation Results 

**Figure 6:**
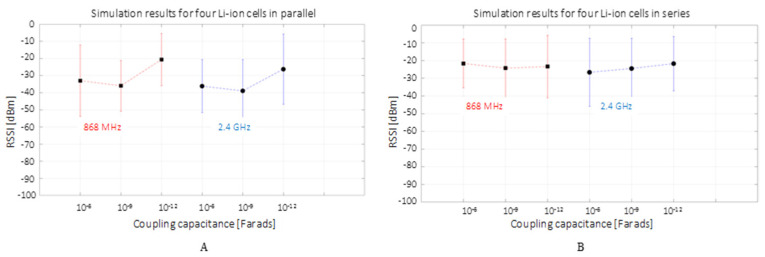
MATLAB simulation received signal strength (RSS) for 868 MHz and 2.4 GHz communication over the DC bus composed of four Li-ion cells organized in parallel (**A**) and in series (**B**). The square marker shows the average measurement interval value in the context of the min–max range bars.

The MATLAB simulation results are illustrated in Figure 6 for the four cells in parallel (A) and series (B) configurations. It can be observed that in both graphs, the RSS presents a relatively large variation between a 0 and −50 dBm interval. This variation, alongside the spectrum analyzer settings, may be accounted for by the discrete components and simulation solver tolerances, the floor function used in the FSK modulation threshold switching, the short simulation time and limited discrete filtering points used in the digital Gaussian filtering. The reception average values show a good reception above −40 dBm in the cell parallel setup and above −30 dBm for the series setup. This indicates a slightly better transmission channel when there is less of current and a higher voltage, as opposite to the case of a low voltage and high current. Furthermore, a better reception is suggested for both frequencies of 868 MHz and 2.4 GHz when the coupling capacitance is on the picofarads order for the high-current scenario, while for the high-voltage setup, the capacitance influence looks similar with an average variation of up to 5 dB for all three capacitive ranges. To summarize, the MATLAB simulation suggests a good high-frequency DC PLC, supporting the decision of empirical scenario testing.

### 3.2. Empirical Experimental Results

**Figure 7:**
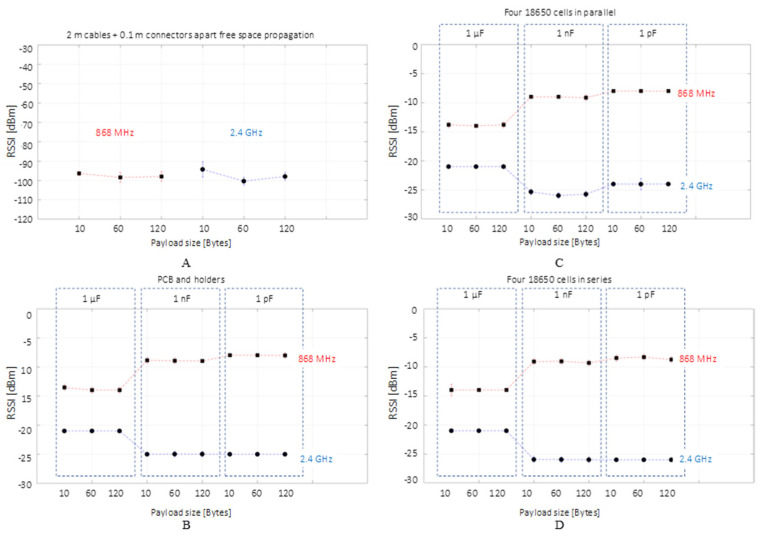
Experimental measurement’s received signal strength indicator (RSSI) for the 868 MHz and 2.4 GHz communication over the 2.1 m separation between the two RF transceivers (**A**), connected to the capacitive coupling PCBs (**B**), linked to the DC bus composed of four Li-ion cells organized in parallel (**C**) and in series (**D**). The square marker shows the average measurement interval value in the context of the min–max range bars.

The results obtained for the four distinct empirical tests are plotted in Figure 7. The 0.1 m coaxial end-cable leakage at 868 MHz and 2.4 GHz shows a valid wireless communication that is possible towards the Rx sensitivity end-value since the BER and PER are 0. On the other end, the second scenario connecting the Tx and Rx only through the capacitive coupling PCB, indicates a stable communication at both frequencies, with a higher attenuation for the 2.4 GHz data. The attenuation for the highest frequency and data rate is expected since, in radio communication, a higher frequency and data rate means a shorter area coverage due to greater attenuation. The highest signal attenuation is not related to the message payload, but to the coupling capacitance; as the capacitance increases, the smaller the attenuation for 2.4 GHz, whereas for 868 MHz, the result is the opposite. When comparing the Li-ion parallel and series scenarios, the RSSI attenuation is similar for both cases with the exception of the 1 pF coupling, in which a variance of approximately 6 dB is recorded accordingly for the 868 MHz and 2.4 GHz transmissions. Compared to the simulation results that indicate a higher attenuation in the presence of a high current for the parallel scenario, the experimental results account for the phenomenon omitted in the simulation: at high frequencies, the wires conduct the signal through their surfaces rather than the volume, with a high proportion of the signal propagating on the surface and around the wire rather than inside it.

Since the RSSI value for the 1 nF and 1 pF intervals experiences approximatively the same signal variation, the overall system’s cut-off frequency behavior is observed when compared to the 1 µF measurements. It acts similar to a low-pass filter for 868 MHz and as a high-pass filter for 2.4 GHz. The *RC* lumped element simulation cannot explain the behavior, since the results indicate the same tendency for both tested frequencies. Furthermore, the occurrence of this relationship cannot be attributed to the cable positions interconnecting the system or to the individual battery holders, since their relative movement when interchanging setups during the experiments did not lead to different results. The phenomena relate to the coupling PCB design since, at microwave frequencies, such as the ones investigated in the present study, the microstrip and strip-line filter theory may provide the answer for the unusual cut-off frequency.

In summary, the empirical tests confirm the simulation suggestion that the high frequencies of 868 MHz and 2.4 GHz provide a stabile communication link over a short-range wired application, such as in the case of DC PLC.

## 4. Conclusions and Further Work

This work showed a successful investigation, conducted through simulation and proven via empirical testing, of high frequency DC PLC. It demonstrated that a short-range wired environment can sustain a stable communication link through 868 MHz and 2.4 GHz at the highest IEEE 802.15.4 standard admissible data rates of 150 kbps and 250 kbps, respectively. Moreover, it showed that communication via interrupted wiring with an end-to-end gap of 0.1 m is also achievable close to the transceiver highest receiving sensitivity; therefore, both planned wired and unplanned wireless (e.g., accidental due to a wire cut or disconnection) communication are possible if RF transceivers are used for DC PLC. This demonstrates that the usage of RF transceivers on short-wired data links can mitigate network discontinuities, ensuring a higher reliability and possible support of new functions, such as communication failure point diagnosis and path estimations. 

This work demonstrates the advantages of using RF transceivers in short-range wired applications, which has a significant impact on the development of current and future BMS technology.

## Data Availability

The dataset generated and analyzed during this study are available from the corresponding author upon reasonable request, but restrictions apply to the commercially confident details.
